# Advancing system and policy changes for social and racial justice: comparing a Rural and Urban Community-Based Participatory Research Partnership in the U.S.

**DOI:** 10.1186/s12939-016-0509-3

**Published:** 2017-02-21

**Authors:** Carlos Devia, Elizabeth A. Baker, Shannon Sanchez-Youngman, Ellen Barnidge, Maxine Golub, Freda Motton, Michael Muhammad, Charmaine Ruddock, Belinda Vicuña, Nina Wallerstein

**Affiliations:** 1 Bronx Health REACH, Institute for Family Health, New York, USA; 2 School of Public Health and Health Policy, City University of New York, New York, USA; 3 College for Public Health and Social Justice, Saint Louis University, St. Louis, USA; 4 Department of Political Science, University of New Mexico, Albuquerque, USA; 5 Men on the Move, Saint Louis University, College for Public Health and Social Justice, St. Louis, USA; 6 School of Public Health, University of Michigan, Ann Arbor, USA; 7 Center for Participatory Research, University of New Mexico, Albuquerque, USA

**Keywords:** Community-based Participatory Research, Social Justice, Chronic Disease, Latino/Hispanic, African American

## Abstract

**Background:**

The paper examines the role of community-based participatory research (CBPR) within the context of social justice literature and practice.

**Methods:**

Two CBPR case studies addressing health inequities related to Type 2 Diabetes and Cardiovascular disease were selected from a national cross-site study assessing effective academic-community research partnerships. One CBPR partnership works with African Americans in rural Pemiscot County, Missouri and the other CBPR partnership works with African American and Latinos in urban South Bronx, New York City. Data collection included semi-structured key informant interviews and focus groups. Analysis focused on partnerships’ context/history and their use of multiple justice-oriented strategies to achieve systemic and policy changes in order to address social determinants of health in their communities.

**Results:**

Community context and history shaped each partnership’s strategies to address social determinants. Four social justice approaches (identity/recognition, procedural, distributive, and structural justice) used by both partnerships were identified. These social justice approaches were employed to address underlying causes of inequitable distribution of resources and power structures, while remaining within a scientific research framework.

**Conclusion:**

CBPR can bridge the role of science with civic engagement and political participation, empowering community members to become political agents who integrate evidence into their social justice organizing strategies.

## Background

Community-based participatory research (CBPR) is a recognized research approach that brings together a diverse array of individuals and organizations that can be used to address the unjust distribution of social determinants that are consistently identified as contributing to health inequities, [1–9]. Principles of CBPR include, building on strengths and resources in the community; facilitating collaborative and equitable partnerships; engaging in power-sharing processes that attends to social inequities; fostering co-learning; and capacity building among all partners [10, 11]. CBPR also combines research and community organizing in ways that contextualize health inequities and create processes that can improve *distributive and procedural justice. Distributive justice* is commonly defined as the right to equal treatment and equal access to the same distribution of goods and opportunities as anyone else [12]. In environmental justice work, distributive justice often refers to efforts to address disproportionate exposure to pollutants and environmental hazards. In public health, distributive justice highlights the need to redress disparate access to resources, assets and services within communities [13–17]. Procedural *justice* refers to the right to equality and democratic inclusiveness in decision-making processes. Of particular importance is the concept of agency, and that community participation and representation in the political processes is key to policy and social change [15, 18–20].

While the current literature underscores how CBPR promotes distributive and procedural justice, [15] there has been less discussion about how CBPR can be used to integrate health concerns into a broader social movement agenda to alleviate social, racial, and economic injustices. Conceptual frameworks from sociology and political science can be useful in exploring how social justice and social movements can enhance our understanding of CBPR’s potential to reduce health inequities [21, 22]. In particular, these frameworks suggest that CBPR partnerships can function as social movements because they explicitly mobilize individuals and organizations to alter power deficits and effect social transformations for sustained community and political action [21, 22].

This social movement/social justice perspective adds to CBPR in that it not only calls for equal access to resources (distributive justice) and equitable voice (procedural justice) but adds two further goals: *structural justice or redistribution* of resources and wealth, and a call to address *politics of recognition or identity politics* [23]. *Structural justice or redistribution* intervention strategies extend beyond those used for distributive justice that often focus on distributing benefits or goods fairly, for example allocating vouchers for farmers’ markets. Structural strategies include transforming broader economic structures such as a fair minimum wage; or democratizing how investment decisions are made, such as tax incentives that enable supermarkets to invest in poor communities, thereby reducing food deserts. While these policy changes might appear to be more nationally based, much of the responsibility for enacting and implementing them occur at the community, locality, or state level.

In contrast, *strategies addressing recognition or identity politics* target injustices that are based in cultural or social identities rooted in domination, and subjected to patterns of communication that are associated with a more privileged social identity. These strategies also target *non-recognition*, or being rendered invisible by authoritative institutions, and disrespected, such as being routinely disparaged in stereotypic representations or everyday micro-aggressions. Remedies for this form of injustice also include revaluing the cultural practices of marginalized groups, gaining recognition of new social identities, and transforming dominant cultural patterns.

CBPR can align with these four social justice strategies (distributive justice, procedural justice, structural justice, recognition/cultural/identity) by using research data to support collective action to change practices and policies through agenda-setting, shaping legislative content, and influencing regulatory policies affecting marginal groups. In addition, incorporating all four social justice strategies highlights the importance of using intervention strategies that place individuals and their pertinent institutions within social, cultural and historical contexts; and encouraging real engagement at all phases of the research process [11, 24, 25].

This paper explores how two CBPR partnerships successfully took a social justice research and action approach combined with their local historic, political, and racial contexts to address inequities and racism, while still staying within a scientific research framework. We analyze how partnerships use multiple justice-oriented strategies to achieve intermediate systemic and policy outcomes. The focus is on these intermediate systemic and policy outcomes given their importance in contributing to behavioral and other health status outcomes.

Both partnerships were initiated in the late 1990s, under the Clinton-era *Conversation on Race,* [26] and received multiple years of funding from the Centers for Disease Control and Prevention (CDC), plus subsequent funding from the National Institute on Minority Health and Health Disparities (NIMHD). Bronx Health REACH (BHR) is a partnership with the Institute for Family Health (a federally qualified health center network), churches, and other community-based organizations collaborating to eliminate health inequities related to diabetes among African American and Latinos in the South Bronx, New York. Men on the Move (MOTM) is a community-academic partnership in rural Pemiscot County, Missouri addressing individual, environmental, and social determinants of heart disease among African Americans. Both CBPR case studies are first presented with attention to how the unique social and political contexts enhance or inhibit the prospects for mobilization, cause particular claims to be advanced rather than others, and impact the strategies that partnerships use. Each partnership is then examined through the four social justice strategies. Results are presented with quotations reflecting partnership achievements and illustrating how differences and similarities in context shaped each partnership’s strategies for social justice, advocacy and interventions to address social determinants of health. We conclude with discussion on lessons learned and implications for future CBPR research and social justice initiatives focusing on reducing health inequities.

## Methods

The two case studies were selected from a larger National Institute of Health (NIH) investigation, led by the University of New Mexico (UNM), [27, 28] to test a CBPR conceptual model; assess the variability of CBPR partnerships nationwide; and identify associations between contexts, partnering characteristics, research, and health outcomes. This mixed methods study [29] consisted of two concurrent research phases: 1) internet surveys to federally-funded research partnerships; and 2) in-depth case studies with academic-community CBPR partnerships. The case study arm used a purposeful sampling strategy to recruit diverse CBPR partnerships: by geographic distribution (both regional and urban/rural); by ethnic/racial or other disadvantaged populations; and by health condition. This paper focuses on qualitative data collected and analyzed for two in-depth case studies.

### Data collection

In 2012, UNM researchers collected data for both academic-community CBPR partnerships. Data collection per case study included: 12–18 semi-structured individual interviews; 1–2 focus groups; a brief close-ended survey distributed to a wider group of partners; document review and a historical timeline exercise with case study partners. Community coordinators facilitated data collection by providing access to partners and to partnership meetings. In keeping with CBPR principles, we developed agreements with each partnership, returning interviews to all study participants, and narratives (with de-identified quotations) to enable co-interpretation and use of the data.

### Data analysis

The analytic process consisted of coding and iteration of transcribed interview and focus group materials, using AtlasTi. Four members of the UNM study team took the lead for each case study, with each member reading and coding transcripts independently, meeting to ensure consistency in coding and theme development, and developing narratives to send back to each partnership. As we completed narrative documents for the two case studies included here, we recognized the potential for mutual learning around CBPR policy change. Thus, we invited members of the BHR and MOTM partnerships to join together on a publication exploring their experiences with a CBPR social justice-oriented partnership engaging in interventions and policy change. For almost 2 years, the group held conference calls and in-person meetings to analyze the data and produce the first draft of the manuscript. The analyses of partnership contexts grounded the analyses of the other themes, as we examined how socio-economic and historical conditions can impact partnership strategies and effectiveness (e.g., how federal or state/local policies, which often maintain discriminatory conditions and fostered research mistrust, can be balanced by social-political community strengths) and community’s history of organizing (e.g., capacity to participate in advocacy which can affect the trajectory of the research). Subsequent analysis focused on partnerships’ utilization of the four types of justice strategies.

### Two case studies

#### The Bronx Health REACH Coalition

With initial funding provided by CDC since 1999, the Coalition with over 70 community and faith-based organizations is dedicated to eliminating racial disparities in diabetes outcomes in the South Bronx and surrounding communities [30]. The South Bronx is the poorest urban congressional district in the United States, where 95% of the residents are African American or Latino, who suffer disproportionately from diabetes with approximately 16% of residents diagnosed, compared to 12% Bronx-wide, 9% in New York City (NYC) and national rates of less than 8% [31, 32]

In 2001, BHR created a Faith-based Outreach Initiative (FBOI) to expand Bronx churches and clergy capacity to integrate information about health disparities and health promotion into their liturgy, implement wellness programs and engage in system-wide changes to address diabetes-related disparities [33]. In 2005, BHR received funding from NIMHD to evaluate the capacity of FBOI: 1) to change knowledge, attitudes and behaviors about healthy eating, physical activity, diabetes management, and navigating the health care system; and 2) to mobilize clergy and congregants to promote access to equitable health care services and healthy food through public policy. A community research committee of residents, community leaders, pastors, physicians, and academics guided FBOI activities and evaluation [34].

Over the years, the FBOI successfully engaged hundreds of community members in diabetes prevention programs promoting active living and healthy eating, resulting in weight loss [35, 36]. At the policy level, BHR launched successful school-based wellness initiatives, resulting in a policy to replace whole milk in all 1579 NYC public schools [37] and passing City Council legislation to further ensure that public school students receive the state-mandated physical education [38]. Many pastors also advocated from the pulpit, [39] mobilizing community members to confront segregated and disparate access to specialty medical care. The result was the filing of a legal complaint with the New York State (NYS) Attorney General against a number of New York City academic medical institutions [40]. Later on local Bronx state elected officials also sponsored a health equality bill in the NYS Assembly and Senate seeking to integrate outpatient specialty care services in NYS teaching hospitals [40]. Most recently, BHR and other partners (including the Bronx Borough President’s Office, the Bronx District Public Health Office of the NYCDOHMH, Montefiore Medical Center, CUNY’s Institute for Equity at Lehman College and other stakeholders) launched #Not62-The Campaign for A Healthy Bronx. This was in response to the Robert Wood Johnson Foundation’s County Health Rankings Report, that ranks the Bronx last among New York’s 62 counties in both health factors and outcomes, including diabetes, infant mortality, and mental health [41]. The #Not62 campaign is a community call to action to its elected leadership, city and state government, business and faith-based leadership, healthcare executives and community residents to build a foundation and infrastructure that address the social and economic factors impacting the overall quality of life. The intended goal is to create an environment that promotes health equity and eliminates health disparity.

#### Men on the move

A rural county located in the Bootheel Region of Southeast Missouri, Pemiscot County is 26% African American, [42] with African Americans in the county having almost double the rate of deaths due to heart disease compared to the state as a whole [43]. About 55% of the African American population has less than a high school education, and 56% live below poverty [42]. Similar to the Bronx, the Robert Wood Johnson Foundation’s County Health Rankings Report ranked Pemiscot County 115th out of 115 counties in Missouri both in regard to health factors and outcomes [44].

The initial work with Pemiscot County started in 1989 through a partnership with Saint Louis University’s (SLU) CDC-funded Prevention Research Center focused on reducing chronic disease. Based on conversations with community partners in 1998, the partnership’s focus shifted from individual risk behaviors to the broader social and environmental determinants of chronic disease. In 2005, the partnership received funding from the NIMHD to co-create Men on the Move (MOTM), a community-academic partnership focusing on educational and economic factors influencing health. Since its inception, the partnership has included community members, community and faith-based organizations, business owners, local governmental leaders, and many others. Including African Americans and Whites, MOTM is unique in a region with a significant history and ongoing individual and institutional racism. Their collective recognition of this served as a “catalyst” to have intentional conversations about racism both institutional and individual, economic deprivation and the impact of these realities on the partnership and the interventions.

MOTM’s initial work, focused on education and economic factors. Their work with education facilitated community members being trained as GED educators and mentors, and the development of GED classes at locations outside the traditional educational system as many of those who needed a GED felt abandoned and ostracized by these institutions. MOTM also facilitated an economic evaluation of the region that led to dialogue and collaborations with local government offices and business leaders and facilitated changes in policies and environments that ultimately expanded job-training opportunities. MOTM also provided a Leadership and Job Readiness course to enhance “soft skills” (e.g., communication, conflict management, team work) among African American men. Those who participated in the course reported increased hope and improved coping post intervention, and approximately 10% obtained full time paid employment [45]. These collaborations also resulted in two local mayors providing land to create production gardens, where the produce was sold to food retailers. In addition, access was granted to city water and permits, previously not available to the African American community. As a result of the individual and environmental level interventions to reduce cardiovascular disease, participants in MOTM reported an increased consumption of fruits and vegetables and decreased hypertension and Body Mass Index [46].

## Results

A combination of 2 focus groups and 28 key informant interviews were conducted. BHR had 23 participants and MOTM had 12 participants (see Table 1 for characteristics of study participants). BHR participants were mostly women (74%) and MOTM participants were equally distributed by gender (*n* = 6 males and 6 females). Participants in the BHR case study self-identified as African American/Black (39%), Latino or Hispanic (26%), and white not of Hispanic origin (35%). Participants in the MOTM case study self-identified as African American (42%) and White not of Hispanic origin (58%). Both groups included participants who were community members and University or Academic partners for the study. BHR participants included 9 University/Academic partners (39%) and 14 Community members (61%). A similar percentage break down of partners’ identities was found in the MOTM group with 4 University or Academic partners (33%) and 8 community members (67%). The study results are presented in two sections: 1) partnership context and participants’ perspectives on their community history and environment and 2) partnership’s social justice approaches.

**Table 1:** Characteristics of participants

	Bronx Health REACH	Men on the Move (MOTM)
	*N* (%)	*N* (%)
	*N* = 23	*N* = 12
Gender		
Male	6 (26%)	6 (50%)
Female	17 (74%)	6 (50%)
Race/Ethnicity		
African American/Black	9 (39%)	5 (42%)
Hispanic or Latino/a	6 (26%)	-
White not of Hispanic origin	8 (35%)	7 (58%)
Partnership Representation		
University/Academic	9 (39%)	4 (33%)
Community Members	14 (61%)	8 (67%)

### Context

Although based in very different socio-historical contexts, both partnerships were acutely aware of the impact of racial segregation and class-based discrimination on social and political conditions. In the Bronx, partners observed the effects of current segregation practices with references to Jim Crow laws. The Jim Crow era, post-civil war, as well as the current New Jim Crow era [47] refers to policies that unfairly discriminate against African-Americans and other people of color, even though they don’t explicitly implicate “race” as the rationale for enacting the unfair laws or ordinances. Bronx partners saw unequal power structures as racialized, with people of color facing discrimination in their day-to-day lives and in access to health care and healthy food.

“So, to me, the disparity is that here we are in 2012 and still there’s a colored door and a White door [to health care]… If you go to the door where you belong, because [you know], that’s where you belong … [then] you know what disparity means … What does disparity mean? It means you can’t have what somebody else has; and so normally, somebody else that has it do[esn]’t look like you. That’s disparity.”

Participants in the Bronx situated current efforts within the historical context of the dearth of public and private resources within medical care. Specifically, partners began to grapple with disparate access to care, focusing on the difficulty in obtaining necessary outpatient specialty care.

“We were absolutely appalled by the fact that we would call for a specialty consult, and if somebody didn’t have private insurance they’d send an intern and then a resident. Then they’d send a fellow, and you were lucky if the person ever saw an attending physician… so we started to really think about what it meant to be operating in a system [of care] within a system [of care] that really was rife with disparities in treatment and how institutionalized this was into the delivery systems that we were interfacing with.”

Community members explicitly linked poor nutrition with a lack of healthy food choices in local markets highlighting the importance of efforts to improve environmental conditions and redistributive policies.

“When we put together that action plan to address obesity, we had discrete programs. Right? We were going to do nutrition education in churches and in an after school program…And as we did the nutrition education, the folks who were getting the nutrition education said, “But it’s not enough to give us this education if when you go outside and you look in our grocery stores you don’t see the food that you’re telling us we should be eating.”

In MOTM, partners understood the unanticipated discriminatory effects of outlawing segregation in a rural environment with already constrained economic opportunities.

“I think in terms of context that is important … is when segregation was outlawed, both in schools and in businesses and other things, what happened was this community lost their African American middle class, because all of the teaching positions went to Whites in power. All of the jobs … it’s still to this day.”

The long-term impact has been the creation of local policies and structure, through informal networks of business interests who have influence over city and county policy, which create significant economic inequities.

“There’s agribusiness down here that has power over and above anything else; and the extent to which they can keep some of these structures hidden enables the power structures to maintain their power. And I think, as a result, a lot of times people who haven’t left the community have absolutely no idea that you could do things differently … There are policies down here that are clearly against federal policy. Whether or not it is brought to light as such, they actually try not to bring it to light.”

Practices of structural racism influence not only the way that the White community interacts with the African American community, but also the way African American community support African American businesses.

“If you put one crawdad in a bucket, you have to put the lid on. [if] you put two crawdads in the bucket; you no longer have to keep the lid on, because they keep each other down. And that is the description that several community members say unbeknownst to each other, years apart they used that same analogy. And it is very, very sad that people in the community don’t support each other’s growth; and so, as a result, they keep each other down. That’s a huge contextual piece in terms of trying to create change in the community. Because any kind of benefit that somebody has, somebody else wants to take it away.”

MOTM grounded their multi-pronged efforts to incorporate behavioral change within the broader scope of these economic and political factors.

“We need jobs. And if I had a job, then I could worry about having enough time to cook a meal or eat this food. But they don’t identify maybe their access to food or their high diabetes or cardiovascular rates as a problem or a need; when they maybe just want jobs or they are struggling with institutional racism, or a sense of hopelessness. I think the project serves as a catalyst to begin those conversations … We have created spaces to have these conversations with the community at the same time as providing access to food and cooking demonstrations.”

### Strategies: social justice approaches

The four strategies are presented through the lens of social justice approaches, as described in Table 2. We have grouped the quotations as primarily representing each strategy, although they often represent multiple forms of policy advocacy and are linked to each other. These quotations demonstrate how different local contexts described above have influenced the partnerships’ choice of social justice strategies to create policy change, and that often these different strategies operate in iterative fashion. The first quotations represent strategies of “identity/recognition” and “procedural justice,” which situate actions within internalized perceptions of community and increased community participation in the political arena. We then present quotations related to “distributive justice” and “structural justice” as more externally-targeted strategies.

**Table 2:** Typology of social justice strategies

		Identity/Recognition	Procedural Justice	Distributive Justice	Structural Justice
Context and History		Injustices that are cultural and are presumably rooted in cultural and social identity domination, non-recognition, and disrespect.	The right to equality in decision-making processes.	The right to equal treatment and equal access to the same distribution of goods and opportunities as anyone else has or is given.	Redistribution of resources in order to address groups’ disadvantages and oppression and to promote equity.
Bronx Health REACH	- City experienced economic and racially charged crisis in the 1960’s- 70’s. - Poor access to healthy food in the community. - Segregation Practices in Health Care delivery.	- Civil rights activism. - Faith-based social movement.	- Representation of communities of color in decision-making/policy bodies. - Faith-based community organizing.	- Provision of fresh fruit and vegetables in all stores, regardless of neighborhood. - Equal access to healthcare regardless of insurance status.	- End Medical Apartheid/Segregated Care. - #Not62 Campaign.
Men on the Move	- Historic rural segregation. - Poor access to healthy food in the community. - Economic depredation.	- Reclaim community gardens/agriculture from slave legacy. - Redefining African American men’s role in the community.	- Representation of communities of color in decision-making/policy bodies. - Challenge race and power structures through vertical alliances with political elites and developers to address economic inequities.	- Increased access to healthy foods in local stores, farmers markets, and through community and production gardens. - Incremental reforms to increase job opportunities for African American businesses.	- Fair wage policies. - Land and water rights.

#### Identity/recognition

Similar to identity-based movements, MOTM and BHR adopted strategies aimed at altering the self-conceptions of disadvantaged groups and challenging negative perceptions of African Americans and Latinos by others. Through the lens of identity and recognition, both groups challenged dominant cultural codes and raised questions regarding how local communities ought to deal with difference.

BHR emerged in a political and social climate where there was longstanding, widespread civic collective action aimed at reducing racial and economic injustices. Black churches, which were deeply entrenched in community organizing dating back to the civil rights movement, served as key leaders in BHR’s efforts.

“I think I got very connected to this vision that I had of health care disparities as a civil rights issue, basically. I kept talking about it as a civil rights issue. People of color are so much more likely to be uninsured or publicly insured than White people, if you discriminate based on the types of insurance people have, which is how the system works in NYC.”

The churches were critical for two reasons: they maintained networks of trust, and they had an infrastructure that supported the group efforts. They were described as the nucleus for the early growth of the project, and the glue that helped mobilize residents.

“We had a member of a church and this member had a sense of ties to the community at large and to the faith-based community [in the Bronx]. And through that member, one of things we decided was to look at faith-based organizations that in many of our communities represent some of the major infrastructures, some of the pillars in that community.”

BHR based their organizing on the values and traditions found in religious faith and commitment to social justice, as well as a commitment to caring for the health of the body within a spiritual framework.

“Well, they made it clear from the jump start that if the community was going to experience wholeness that the church, and particularly the Black and Latino churches, must see health issues as a spiritual matter. Now, when they made it spiritual, that’s what got my attention. They are willing to address this from a biblical and a theological perspective. I had the tools to do that. And with the background and appreciation for liberation theology, I began to see it through the eyes of faith and theology. God created us to be healthy. And God desires that we be healthy and prosperous, spiritually, physically, mentally and emotionally… If they’re separated then you do damage to the whole soul. And that’s one of the things that convinced me that I needed to be involved.”

An impetus for MOTM was a community conversation in which an African American man said he felt “unwelcome at community events”, and participants reflected on the social, political and economic reasons why there were so few male role models in the community. MOTM worked to involve men who have been otherwise disempowered, to redefine African American men’s roles, and to reclaim ownership of agricultural work as a way to rebuild community. One community partner suggested “initially, African American men frowned upon other men with hoes working in the garden … relating it back to slavery.” However, as gardening groups gained presence in the community, and people saw that the program was hiring above minimum wage, painful associations with slavery were diminished, and community norms started to change.

“We’ve got guys the past couple years that worked in the garden that … [were on] parole or felon. We hire them. It helps to bring more people in, because these are guys that come off the street … well, man, they gave me a chance … [You have to] really take in community … You just can’t take in people that are doing well. You have to take in people that’s having a hard time. You can’t leave anybody out.”

Moreover, the visibility of MOTM’s agricultural work modeled civic engagement that facilitated a shift in the perspective of the broader White community, from non-recognition and disrespect of African American men to acknowledging them as a valuable part of the community.

“We were working in the ballpark-that’s where one of our gardens is. The ballpark shed needed to be cleaned out. Frank offered to this gentleman, who runs the ballpark, to clean out the shed with some of the guys from Leadership and Job Readiness. And he was very hesitant-the ballpark guy-but he allowed them to do it; afterwards, he came over and took them all out to breakfast, and he said how grateful he was; and he never would have actually believed that they could have done it … A lot of the Whites have never interacted with African Americans in this community … Those little tiny successes are huge in this community.”

Though not named a faith-based initiative per se, MOTM also drew from the important role that the church plays in the Bootheel community.

“Using the churches in the community to help spread the word, to help get people in the church. Get your family. Set up a planting day … It’s called, “Plant a Row.” … Say you want to plant a row of beans besides your house and have a Men on the Move sign…you and your friend will get together plant a row, we’ll take half, give back to the community; the other half go to you and your family.”

#### Procedural justice

By its very nature, CBPR seeks to advance procedural justice by incorporating community members and disadvantaged groups as central players in both research and policy advocacy. The MOTM and BHR partnerships pursued different strategies to achieve adequate representation and voice among community members. In the South Bronx, participants noted that key leaders in the current project have been involved in long-term organizing to revitalize the area after the economic and social crisis of the late 1960’s and 70’s.

“Some of the [BHR] leaders were part of a very big movement in the Bronx in the ’80s to help rebuild the Bronx after it had been really gutted by arson and greed. These are people out of the community who wrestle to the forces, wrested the Bronx from those forces, [and] cleaned up the community. Where you had mattresses and crack vials and drug needles, those people worked to create apartment buildings, home ownership, built schools, started afterschool program[s]. Those are the people who are the leaders and the foundations of our work.”

Not only were churches seen as deeply entrenched in community organizing in the Bronx, they also cultivated active ministries that supported community leadership, itself a tenet of CBPR capacity-building.

“So that’s why we talk about this as faith-based … whether it’s in the nurses’ ministry, the men’s ministry, the women’s ministry, the children’s ministry … we’ve had programs that have come organically out of the church, where a member of that church has created or developed a [health] program that we, then, with the resources that we have been able to sort of codify those programs”

MOTM did not have organized structures to build on, yet successfully developed strategies to convince local developers and political elites to work with them to address structural economic inequities. MOTM used the privileged position of some core (White) university team members to make the community more visible and challenge traditional racist lens. This process of gaining access to White power structures began to democratize policy decisions, creating new avenues for longer-term reforms.

“What can we do to address the problems? All sorts of things … we’ve found that having the academic presence there, which for better or worse [is] primarily a White presence, helps people … and at one point we had a White businessman partner who was with us who helped to talk to a White economic developer; and that helped to pave some pathways that we now can walk on. So we sometimes create pathways that enable decisions to be made in our favor. Again, it’s very racially biased. And we continue to try and get access to those power places outside of our own little world with variable success.”

As part of developing collective capacity, the MOTM partnership conversations modeled democratic decision-making and promoted a sense of power in all partners. Participants listened to the experiences and knowledge of the community as a strategy for recognizing community voice.

“Here we’ve got local knowledge in the community. They have a good sense of what works best, what has worked best in past years … versus another level of experience coming from outside of the community … For community members that don’t know the value of their voice, sometimes you see them not wanting to speak loud so they are heard, when it’s so important that they speak loud. So that the university partner, you could say, hears them.”

Finally, increasing rural community members’ exposure to national networks was a key component of the organizing process as it facilitated capacity building and community leadership.

“My involvement with NCC [National Community Committee of the CDC Prevention Centers] made me aware of the role that community has in addressing these social issues, the diversity in strategies and degree of engagement. It has also helped me to see clearly next steps in the process to accept responsibility as a part of the community and empower others to play an active role in helping to address these issues. I feel strongly that MOTM has laid a foundation for the social justice movement to take root in Pemiscot County.”

#### Distributive justice strategies

Demands for recognition and voice in democratic processes were intertwined with efforts to engage in distributive justice strategies to increase access to resources that had been previously denied them. For example, BHR specifically undertook increasing access to healthy food, including working with churches to “adopt a Bodega.”

“You keep telling us to eat [healthy], but we don’t have any place to shop. The bodega on my corner doesn’t have low-fat milk or 1% fat milk. They don’t have vegetables. They don’t have fruit…So we needed to talk about how we can look at these bodegas in the community and see how we can influence [them] to change some of the kinds of products and produce that they sold.”

BHR partners also became aware of barriers to health care, through attempting to provide care to individuals in their congregations.

“I had one of the young men in the choir. He was passed out in the choir one Sunday. And everybody went running to him…But he says, ‘No, no, no… Don’t call the ambulance. Don’t call the ambulance.’ ‘What do you mean don’t call the ambulance?’ ‘No, I have no insurance. I have no insurance…I just don’t want them to send me no bills.’ It was instances like these that gave life to the Campaign to End Segregated Care.”

Similarly, MOTM increased access to fresh produce through direct access to community gardens and through production gardens that provided produce to local vendors. One of the things this required was getting access to water for the gardens.

“The mayors in Caruthersville and Hayti helped us to get water to the land. We weren’t able to at first, but they helped both in terms of permission to access the water and they also helped us to build the proper pipes.”

MOTM also worked with local grocery stores to ensure access to low fat and low sodium products.

“We worked with the local grocery stores to be sure they carried low fat and low sodium products. Part of what made this possible was having taste testings so that customers would know how these would taste and how to prepare the different foods. We also used shelf talkers—signs that highlighted low fat and low sodium options.”

#### Structural justice strategies

Both partnerships have used recognition/identity politics, procedural justice and distributive justice approaches to build the foundation for structural justice approaches. The history of civil rights organizing in the Bronx facilitated structural campaigns, and the churches provided the mechanism to build on congregant’s spiritual and civil rights identities to advocate structural changes. Community partners cited a mixture of collective bargaining and conflict to achieve social justice aims “one of the unique things that sort of evolved was that … we weren’t beholden to anybody in the established medical community… We could challenge those things that were not working for us.” Throughout the process, the coalition retained enough independence from the medical establishment to agitate for change through legal mechanisms. After the project pushed for health care reforms through political channels that “went nowhere” they filed a complaint against several hospitals.

“The elected officials previously had been very indifferent regarding this. We went to Albany I think about seven, maybe eight years ago to meet with elected officials on this issue, and we were politely received, as is often the case when you meet with them. But nothing happened. Do you understand? Of course, we filed a [complaint] against these hospitals with the Attorney General’s office.”

**Fig. 1:**
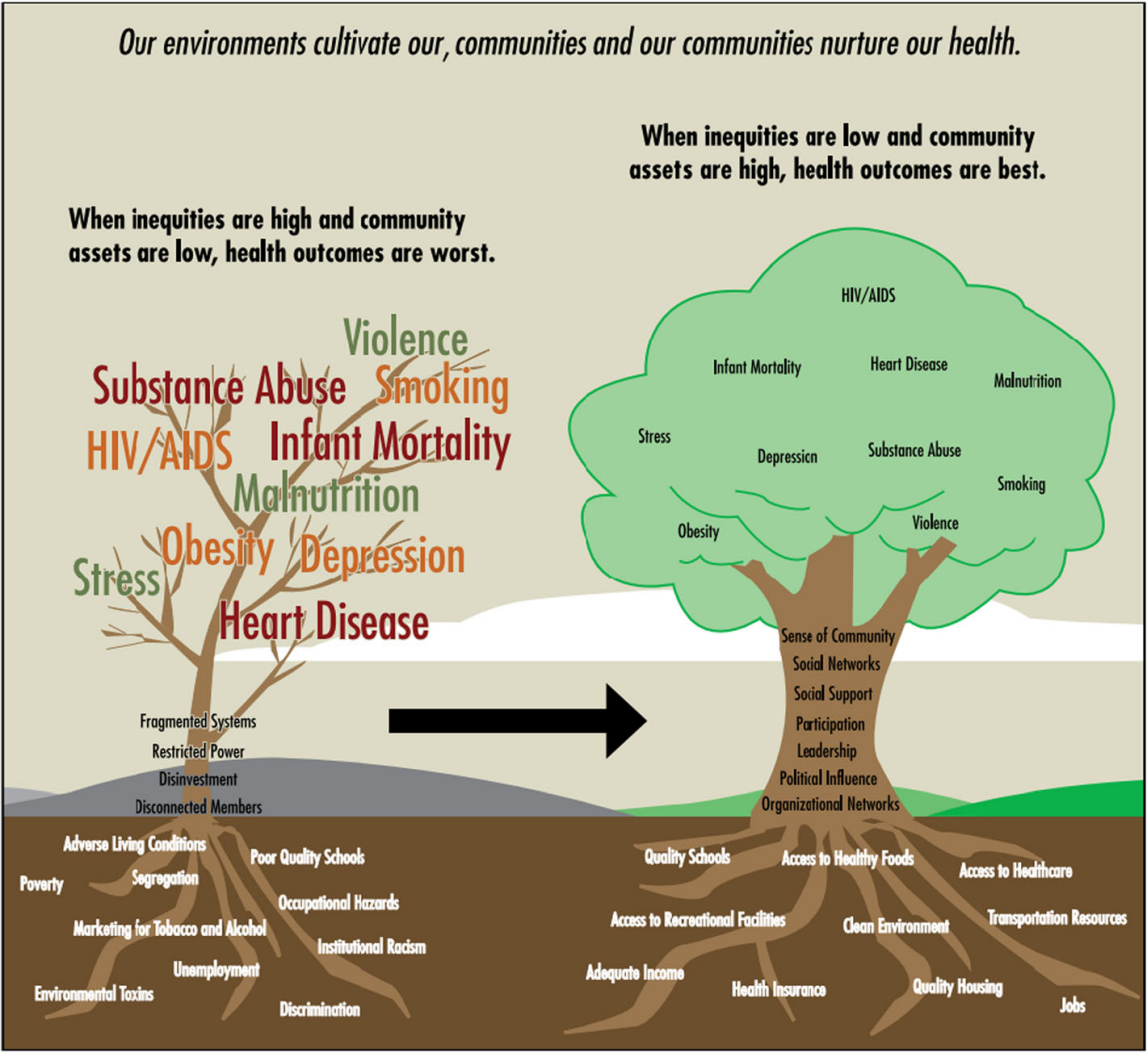
Growing Communities: Social Determinants, Behavior, and Health^a^. ^a^ Published in Brennan RL, Baker EA, Metzler M. Promoting health equity: A resource to help communities address social determinants of health. Atlanta: U.S. Department of Health and Human Services, Centers for Disease Control and Prevention; 2008 (Authors obtained permission to reproduce this figure)

One major step toward structural justice for both partnerships is the acknowledgement that power and privilege varies among community and academic partners. For example, BHR coalition members of color often cited the leadership of a White doctor as validating their experience of discrimination in medical settings. This was also noted in MOTM, with the White PI committed to social justice, where partners made an early decision to address power and privilege differentials in discussions and readings (See Fig. 1). As a result, the partners agreed to reallocate funds to local coalitions and to use the privilege of the academic partners to create a bridge between political elites and community partners.

“There’s some work that we did that was helpful in terms making sure we were on the same page … We have this tree picture that shows two different trees. One tree has heavy disease burden in the branches, and then minimal community supports in the trunk, and then root determinants such as high levels of poverty, high unemployment, and racism. The other tree has lower disease burden, strong community networks and supports, and root determinants such as good educational opportunities and jobs. So we use things like that to start talking about kind of what’s going on. We also read some things together that addressed race and racism … there was one on the experience of being a Black man …we used some of those pieces to have dialogue within our own partnership; to engage people in conversation and restructure our work to go beyond just behavioral factors.”

In addition, acknowledging the lack of job opportunities and hurdles to employment, MOTM focused on economic development and policy actions around land. As an incremental policy reform, MOTM key partners engaged in strategic bargaining with elected officials to foster the growth of African American businesses throughout the area.

“The mayor of Hayti has been willing to give us plots of land to grow on. And we are hoping that we can even now begin to sell our produce from that plot of land which is all of a sudden … We now have a set of customers that’s willing to purchase food from us; and we’ve distributed the food publicly and gotten donations before. But this will be the first time that we’re edging ever so slightly into this business model.”

## Discussion

The case studies presented here suggest that CBPR partnerships can work toward emancipatory change by engaging in struggles for identity/recognition and procedural justice, while simultaneously moving toward distributive and structural justice. This social justice framework underscores the reality that communities do not have compartmentalized problems and that the political, economic and social causes are strongly related [48]. High rates of diabetes and heart disease are not separate from the shortage of jobs, limited access to fresh fruits and vegetables, and the lack of health insurance. Neither do these problems have compartmentalized solutions. Jagosh et al. [49] found, that partnership synergy, or the bringing together of multiple partners (and hence skills and perspectives) can lead to positive outcomes (e.g., trust, enhanced data collection) as well as form the foundation from which broader capacity and systems change outcomes emerge (e.g., policy and organizational changes). Presented here are four of the major lessons learned from our work highlighting key differences and similarities of both partnerships: 1) the importance of historical understanding of racial and social context, 2) the role of context in shaping partnership social justice strategies, 3) the role of national funding, and 4) the connection between CBPR and social justice movements.

1. *Historical context and recognition of racial and social injustices*

Despite the different historical and social contexts from which these partnerships evolved, there were common reflections about the recognition of racial and social injustices, which help partners build stronger and more trusting relationships. At the same time, the recognition of social injustice promoted partnership synergy as a proximal outcome that was instrumental in achieving the specific project and/or policy outcomes. In MOTM, their project emerged from a context of rural segregation and economic deprivation due to loss of land, jobs and African American owned businesses. The MOTM partnership explicitly engaged in building capacities to reduce economic inequalities through job creation and sustainable agricultural development, while simultaneously acknowledging that it was equally important to attend to more immediate problems such as high rates of diabetes and cardiovascular health. Early in their project, the MOTM partnership understood that unacknowledged racial segregation within rural economically disadvantaged environments intersects with the socioeconomic inequality between Blacks and Whites.

Similarly, the BHR partnership recognized a history of racial and social injustices in the South Bronx early in their project. In the case of BHR, their project emerged from a context of urban segregation, disinvestment in the 70's, and a dearth of services in the area (i.e., housing, medical care, education, access to healthy food). BHR coalition leaders had a long history of civil rights organizing and saw the current project as a part of long-term effort to rebuild the neighborhood. Over the years, the BHR coalition sponsored a range of health promotion activities addressing food access. They also decided to confront the power structures that shape access and delivery of health care in academic hospitals according to type of health insurance that effectively discriminates against poor, racial and ethnic minorities.

2. *Social justice partnership strategies built on context*

Our analysis indicated that while context was important in both case studies, the way these unfolded was different in each setting. Our analysis suggests that differences in social, political and economic context contributed to key differences in the relational dynamics of each partnership and influenced their actions. For MOTM, in terms of procedural justice this entailed strengthening cultural identity, community leadership and capacity building. With regard to distributive justice policy the emphasis was on increasing access to water and land rights and African American employment. In doing so, MOTM pursued incremental policy and economic development strategies to increase the employment of African American males, while simultaneously reclaiming farming practices, historically seen as reproducing oppressive slavery practices and as a transgressive practice of cultural pride. This approach addressed the limited systematic community organizing in the community, and the lack of African American leaders representing the needs of African Americans in Pemiscot County. Access to resources and positions of power were the first and most feasible steps to pursue broader structural changes given the community’s history.

For the BHR group, their connection to civil rights movement enabled them to build from cultural identity and leadership capacity to seek distributive justice targets such as increased access to healthy foods, as well as to advocate for structural change in inequitable health system structures. The BHR partnership recognized poor quality and access to health care in the community and pursued policy reforms aimed at ending medical apartheid known as “the segregated care campaign”. By partnering with churches and community-based organizations, they engaged community members in supporting regulatory and legislative strategies to change the practice of steering patients to separate health care settings based on insurance status. This strategy was aimed at structural redistribution, because it called for new systems of health care, and it was a strategy of identity recognition because it sought to remediate the subordination of communities of color in the Bronx by linking poor health to wider patterns of racial discrimination.

In both cases, CBPR facilitated opportunities for partners to listen to each other and capitalize on power and privilege differences among their members (i.e., White physician, elected officials, academics, pastors, etc.). Furthermore, both groups similarly collaborated with churches to mobilize community members as churches provided the mechanisms to engage community residents through two related worlds: the spiritual and the social justice one.

3. *Role of national funding*

Funding that allows for planning and capacity building in communities proved to be important for these two partnerships to maintain a commitment to promoting a social movement. Both partnerships started with capacity-building funds from the Centers for Disease Control, with subsequent NIMHD research funds supporting their capacities to address racial and social inequities contributing to health disparities. Both funding streams validated community participation as part of their funding requirements. For example, CDC REACH funding awarded to BHR and CDC Prevention Research Center funding awarded to MOTM required that community-based organizations were integral to the grant design and implementation. Additionally, both partnerships received two iterations of the 11-year CBPR pathway, funded by NIMHD, which supported a 3-year planning grant, followed by 5 years of intervention research. Both partnerships shared funding with partnering community-based organizations in order to build capacity and re-energize community advocates and volunteers, which ultimately provided greater potential for change. The longevity of funding also allowed the partnerships to build sufficient trust to process the privilege and power dynamics inherent in community-academic partnerships where traditionally the academic partners dominate.

As indicated in both case studies, federal agencies commitment to long-term funding streams that support continuous partnership development are critical because these can evolve into effective health and racial equity advocacy entities. Long-term funding, coupled with requirements to share funding with communities (or to base funding within community based organizations and tribes as non-traditional grantees) can strengthen the potential of CBPR partnerships to influence procedural justice and/or bolster other forms of social justice articulated in this paper.

4. *CBPR and social justice movements*

Our analysis demonstrates that CBPR partnerships can achieve both short and long-term policy changes aimed at remediating a variety of injustices experienced by communities. A key lesson from our analysis is that building from core CBPR values, a social justice orientation, does not preclude partnerships from effectively achieving specific grant outcomes. On the contrary, our analysis suggests that both the MOTM and BHR crafted multilevel interventions that sought individual health gains as well as working towards achieving multiple forms of justice.

Our study illustrates how CBPR partnerships are capable of pursuing structural policy reforms even under very different social and political conditions. CBPR partnerships can provide an important deliberative function that encourages political participation from groups that are typically marginalized in US democracy. Indeed, our analysis reveals how both projects represent a form of social movement building that can increase civic engagement.

### Study limitation

There are several limitations to our work. Perhaps most important is that almost by definition the outcomes achieved by projects that use CBPR approaches depend to a large extent on the specific context as well as the values and capacities of the partners involved. Therefore, the aspects of the context and partners that were found to be important within our two case studies may have differing importance in other contexts and among other partners. Another limitation to generalizing our work is that our work here engaged African American and Latino communities. The specific challenges faced by CBPR partnerships working with other marginalized groups and the strategies required to redress their injustices will require specific analyses of their own histories, contexts, values, and capacities.

## Conclusion

CBPR, when it is employed within the context of social justice, is far more than a research modality, and can accomplish far more than intended health and grant outcomes [50]. As demonstrated above, BHR and MOTM have fostered social cohesion, built local capacity and helped strengthen leadership within and among community partners—skills that the community will retain long after the projects and researchers are gone. Moreover, CBPR can bridge the role of scientific evidence with civic engagement and political participation, strengthening community members as political and social agents who can integrate data into their social justice and community organizing strategies.

## Abbreviations

BHR: Bronx Health REACH
CBPR: Community-based Participatory Research
CDC: Centers for Disease Control and Prevention
FBOI: Faith-based Outreach Initiative
MOTM: Men on the Move
NCC: National Community Committee
NIH: National Institute of Health
NIMHD: National Institute on Minority Health and Health Disparities
NYC: New York City
SLU: Saint Louis University
UNM: University of New Mexico
